# Modulation of Disease-Associated Pathways in Hidradenitis Suppurativa by the Janus Kinase 1 Inhibitor Povorcitinib: Transcriptomic and Proteomic Analyses of Two Phase 2 Studies

**DOI:** 10.3390/ijms24087185

**Published:** 2023-04-13

**Authors:** Huiqing Liu, Leandro L. Santos, Susan H. Smith

**Affiliations:** Incyte Corporation, Wilmington, DE 19803, USA

**Keywords:** hidradenitis suppurativa, inflammatory skin diseases, proteomics, transcription, INCB054707, JAK1, povorcitinib

## Abstract

Janus kinase (JAK)/signal transducer and activator of transcription signaling (STAT) has been implicated in the pathophysiology of hidradenitis suppurativa (HS). This study evaluated treatment-related transcriptomic and proteomic changes in patients with moderate-to-severe HS treated with the investigational oral JAK1-selective inhibitor povorcitinib (INCB054707) in two phase 2 trials. Lesional skin punch biopsies (baseline and Week 8) were taken from active HS lesions of patients receiving povorcitinib (15 or 30 mg) once daily (QD) or a placebo. RNA-seq and gene set enrichment analyses were used to evaluate the effects of povorcitinib on differential gene expression among previously reported gene signatures from HS and wounded skin. The number of differentially expressed genes was the greatest in the 30 mg povorcitinib QD dose group, consistent with the published efficacy results. Notably, the genes impacted reflected JAK/STAT signaling transcripts downstream of TNF-α signaling, or those regulated by TGF-β. Proteomic analyses were conducted on blood samples obtained at baseline and Weeks 4 and 8 from patients receiving povorcitinib (15, 30, 60, or 90 mg) QD or placebo. Povorcitinib was associated with transcriptomic downregulation of multiple HS and inflammatory signaling markers as well as the reversal of gene expression previously associated with HS lesional and wounded skin. Povorcitinib also demonstrated dose-dependent modulation of several proteins implicated in HS pathophysiology, with changes observed by Week 4. The reversal of HS lesional gene signatures and rapid, dose-dependent protein regulation highlight the potential of JAK1 inhibition to modulate underlying disease pathology in HS.

## 1. Introduction

Hidradenitis suppurativa (HS) is an autoinflammatory skin condition that causes recurrent painful nodules, abscesses, and tunnel formation, leading to chronic non-healing wounds and scarring [1,2]. The pathophysiology of HS is considered complex and is not fully understood, creating challenges in selecting treatment approaches [3,4]. Nevertheless, recent evidence indicates that occlusion and consequent inflammation of hair follicles play a critical role in the development of HS by activating various pathways of skin inflammation [5,6].

Proteomic analysis in a limited number of samples showed that patients with HS had a greater dysregulation of circulating proteins compared to patients with psoriasis, demonstrating a larger systemic burden [7]. HS and psoriasis samples had 17 upregulated proteins in common, including interleukin (IL)–6, IL2 receptor α (IL2RA), IL17A, C-X-C motif chemokine ligand 10 (CXCL10), and CD177. The overexpression of lipocalin 2 appeared unique to HS and may be a biomarker for HS subtypes with a higher inflammatory burden [7,8].

Previous transcriptomic analyses of HS lesional skin have identified several inflammatory pathways that may be implicated in disease pathogenesis [8,9,10]. In an analysis of 144 genes in skin-derived RNA from healthy subjects and patients with mild-to-severe HS, 129 genes were significantly elevated in HS lesional skin compared with skin from healthy controls [9]. Upregulated genes included *IL1A*, *IL6*, *TNF-α*, members of the *IL17* and *IL10* families, members of the *IFN* family, and tyrosine kinases (*JAK1*, *JAK3*, *BTK*, and *SYK*) and their downstream signaling partners (*STAT* family) [9]. In a recent study, both HS lesional and perilesional skin were shown to have inflammatory gene signatures, highlighting that inflammation is occurring beyond visually apparent sites of disease [8]. An enrichment for complement activation and B-cell signaling pathways was also noted in HS. Furthermore, a meta-analysis of RNAseq data from three separate studies confirmed the impact of HS on skin gene expression related to inflammatory pathways, epithelial differentiation, and dysregulated metabolism [11]. Finally, an analysis of two published data sets compared microarray data from HS lesional and non-lesional skin and RNA-seq data from wounded and non-wounded human skin samples [10]. As both HS lesions and chronic non-healing wounds are associated with dysregulated immune responses [12,13], similarities between gene signatures were explored. Study findings demonstrated that HS lesional skin shared a similar gene expression profile with wounded skin, with upregulation of various antibacterial and antiviral genes, and simultaneous downregulation of genes encoding antimicrobial peptides associated with normal sweat duct and skin biology [10].

Several differentially expressed genes (DEGs) identified in the prior analyses are regulated to some extent by the activation of Janus kinases (JAKs) and subsequent induction of signal transducer and activator of transcription (STAT) activity. Targeting the JAK pathway in HS was investigated because the JAK/STAT signal transduction pathways are responsible for the production of numerous pro-inflammatory mediators thought to contribute to the pathology of HS [14,15,16,17,18]. Povorcitinib (INCB054707) is an oral small-molecule JAK1 inhibitor with approximately 52-fold greater selectivity for JAK1 versus JAK2 [19]. In two phase 2 studies, orally administered povorcitinib was generally well tolerated and demonstrated proof of concept in the treatment of patients with moderate-to-severe HS [19]. In this analysis, we evaluated disease biology and treatment-related changes by performing transcriptomic RNA-seq analyses of lesional skin biopsies and proteomic analyses of blood samples from patients receiving povorcitinib or placebo in the two trials.

## 2. Results

### 2.1. Transcriptomics

**Figure 1:**
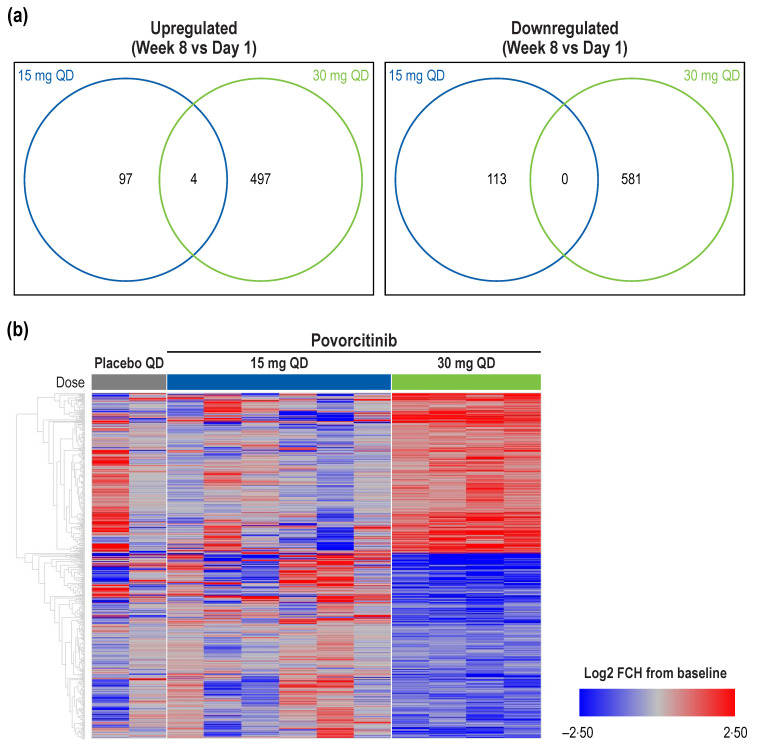
Differential gene expression following treatment with povorcitinib. (**a**) Overlap between 15 and 30 mg povorcitinib QD for DEGs in HS lesional skin biopsies from Week 8 vs. baseline. (**b**) Heatmap of treatment-mediated gene expression change in lesional HS skin biopsies between Week 8 and baseline. DEG, differentially expressed gene; |FCH|, absolute fold change; HS, hidradenitis suppurativa; QD, once daily. The cutoff for significance was *p* < 0.05 and |FCH| >1.5.

Lesional skin punch biopsies from 12 sample pairs taken from the edge of an active HS lesion [20]; both baseline (Day 1) and Week 8 data (15 mg povorcitinib once daily [QD], *n* = 6; 30 mg povorcitinib QD, *n* = 4; placebo QD, *n* = 2) were available for analysis. In RNA-seq analysis of HS lesional skin, there was minimal overlap between the 15 and 30 mg povorcitinib QD groups in DEGs from Week 8 versus baseline (Figure 1a). Consistent with its characterization in the efficacy studies as a potential minimum effective clinical dose [19], the 15 mg povorcitinib QD group had a notably reduced effect on gene expression compared with the 30 mg QD group, as indicated by the lower number of significant DEGs. A total of 1082 DEGs between Week 8 and baseline were identified in samples from the 30 mg povorcitinib QD group (Figure 1a,b). Of these, 501 DEGs were induced and 581 DEGs were repressed. Consistent with clinical response data, placebo and 15 mg povorcitinib QD showed a similar transcriptomic pattern to each other (Figure 1b) [19].

**Figure 2:**
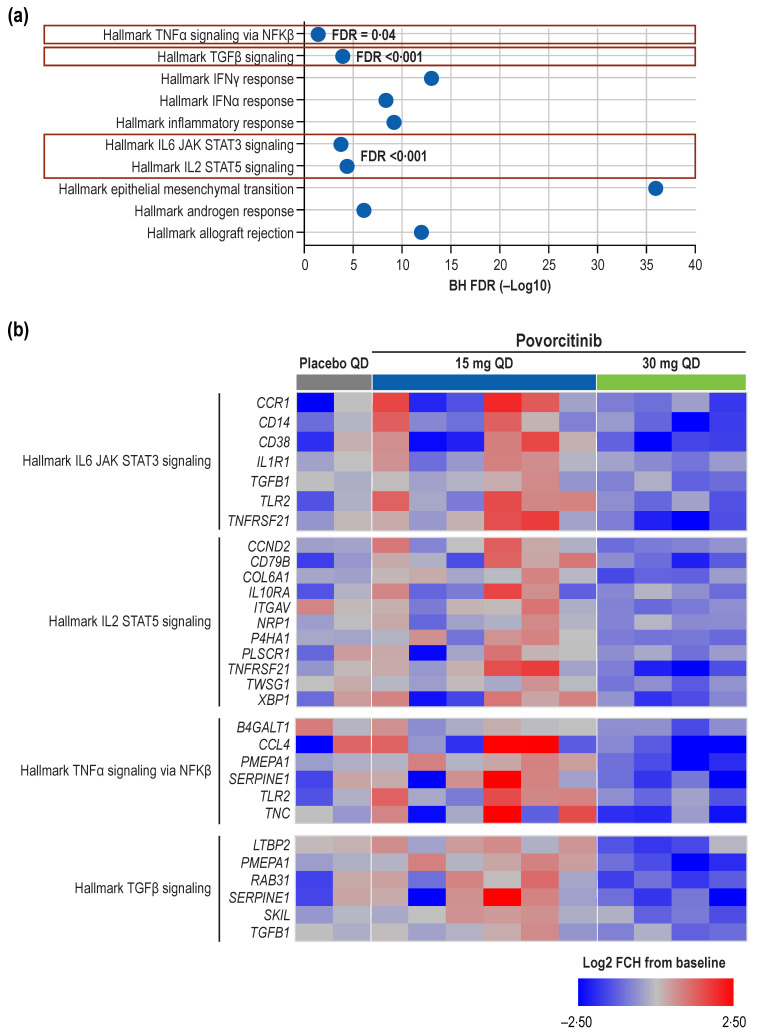
Pathway enrichment analysis. (**a**) Pathway enrichment analysis at Week 8 of treatment with 30 mg povorcitinib QD and (**b**) heatmap of Week 8 DEGs from selected Hallmark signaling pathways. DEG, differentially expressed gene; FCH, fold change; FDR, false discovery rate; QD, once daily. Log2 FCH from baseline is used. HALLMARK signaling pathways were from the Molecular Signatures Database (MSigDB v7·2 [21]).

In the pathway enrichment analysis, a number of inflammatory pathways were found to be enriched in the Week 8 DEGs from the 30 mg povorcitinib QD group (Figure 2a). Specifically, JAK/STAT signaling pathway transcripts (*CCND2*, *CCR1*, *CD14*, *CD38*, *CD79B*, *COL6A1*, *IL1R1*, *IL10RA*, *ITGAV*, *NRP1*, *P4HA1*, *PLSCR1*, *NFRSF21*, *TGFB1*, *TLR2*, *TNFRSF21, TWSG1*, and *XBP1*) were significantly reduced (false discovery rate [FDR] < 0.001; Figure 2b). Genes regulated by nuclear factor (NF)-κβ in response to tumor necrosis factor (TNF)-α signaling (*B4GALT1*, *CCL4*, *PMEPA1*, *SERPINE1*, *TLR2*, and *TNC*) were also significantly downregulated (FDR = 0.04). Finally, significant reductions (FDR < 0.001) in transcripts of genes regulated by transforming growth factor beta (TGF-β [*LTBP2*, *PMEPA1*, *RAB31*, *SERPINE1*, *SKIL*, and *TFGB1*]) were observed.

**Figure 3:**
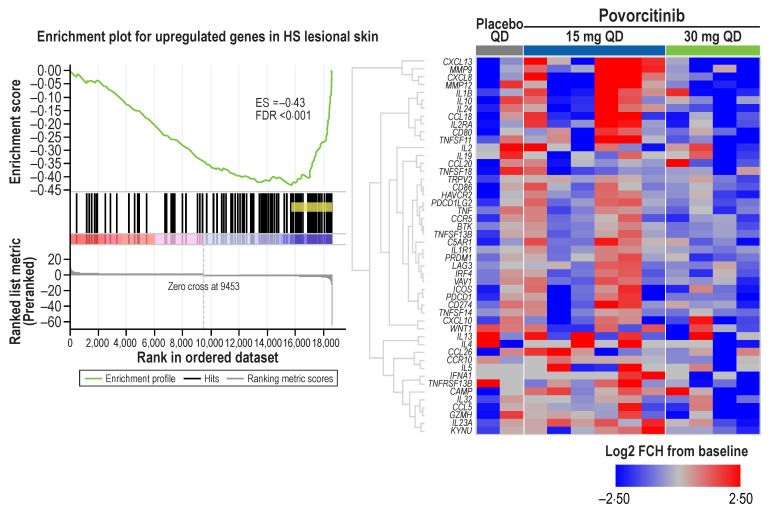
GSEA and enrichment plot showing signature reversal of upregulated genes in HS lesional skin after 8 weeks of treatment with 30 mg povorcitinib QD. * ES, enrichment score; FCH, fold change; FDR, false discovery rate; GSEA, gene set enrichment analysis; HS, hidradenitis suppurativa; QD, once daily. * Plot represents a case (30 mg povorcitinib QD treatment) and GSEA data based on previously described HS gene signature (HS-high) [9]. Genes displayed in heatmap are marked in yellow in the corresponding GSEA plot.

We previously reported on a 128-gene signature of HS [9]. In the present analysis, we evaluated the effect of povorcitinib on this 128-gene signature by gene set enrichment analysis (GSEA). A reversal from the HS disease-associated gene signature was observed upon 8 weeks of treatment with 30 mg povorcitinib QD (Figure 3). Overall, 99 of 128 genes were downregulated by treatment at Week 8, revealing a negative enrichment score (−0.43; FDR < 0.001).

**Figure 4:**
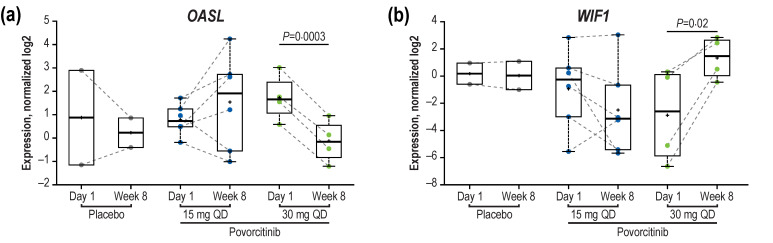
*OASL* and *WIF1* gene expression in HS lesional skin biopsies over 8 weeks of treatment. (**a**) OASL, (**b**) WIF1. HS, hidradenitis suppurativa; QD, once daily.

Treatment-related changes with 30 mg povorcitinib QD were also assessed on two previously published gene sets from HS lesional (vs non-lesional) and wounded (vs non-wounded) skin. The overlap of DEGs in HS lesions with genes expressed during wound healing was previously reported [10]. Using the same data set from the previous study, the gene signatures used for GSEA in the present analysis consisted of 1131 upregulated and 516 downregulated genes in HS lesional and wounded skin (Figure S1). Full lists of genes shared by HS lesional and wounded skin and reversed by treatment with 30 mg povorcitinib QD are shown in Supplementary Tables S1 and S2. At Week 8, a reversal of genes upregulated in HS lesions and wounded skin was observed after treatment with 30 mg povorcitinib QD (Figure S2a), including downregulation of many interferon (IFN)-stimulated genes, IL-6/STAT3 targets (*CCR1*, *CD14/38*, *IL1R1*, *TGFB1*, *TLR2*, and *TNFRSF21*) [21], genes with innate inflammatory and antiviral functions (e.g., *PARP14*, *ISG20*, *OAS2*, and *OASL*; Figure 4a), and genes with antimicrobial properties (e.g., *TDO2* and *DEFB103B*; Table S1). Conversely, genes lowly expressed in HS lesional and wounded skin were upregulated after 30 mg povorcitinib QD treatment (Figure S2b), including genes involved in sweat gland development such as *WIF1* (Figure 4b), *KRT77*, and *KRT31* (Table S2). Finally, *FOXA1*, a transcription factor required for sweat secretion, previously shown to be downregulated in HS lesional skin and upregulated in wound-healing skin, was significantly upregulated following 8 weeks of treatment with 30 mg povorcitinib QD.

### 2.2. Proteomics

**Figure 5:**
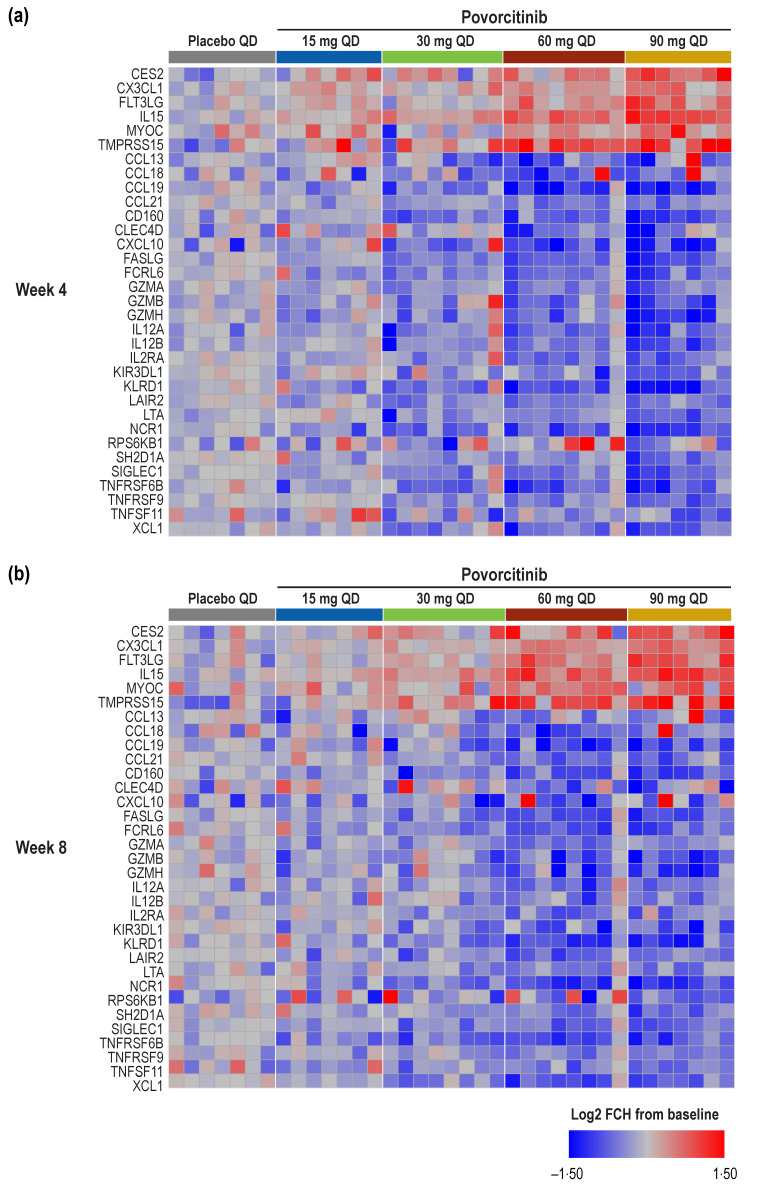
Heatmap of DEPs in blood samples by treatment. (**a**) Week 4, (**b**) Week 8. DEP, differentially expressed protein; |FCH|, absolute fold change; FDR, false discovery rate; QD, once daily. The cutoff for significance was FDR < 0.05 and |FCH| > 1.5.

**Figure 6:**
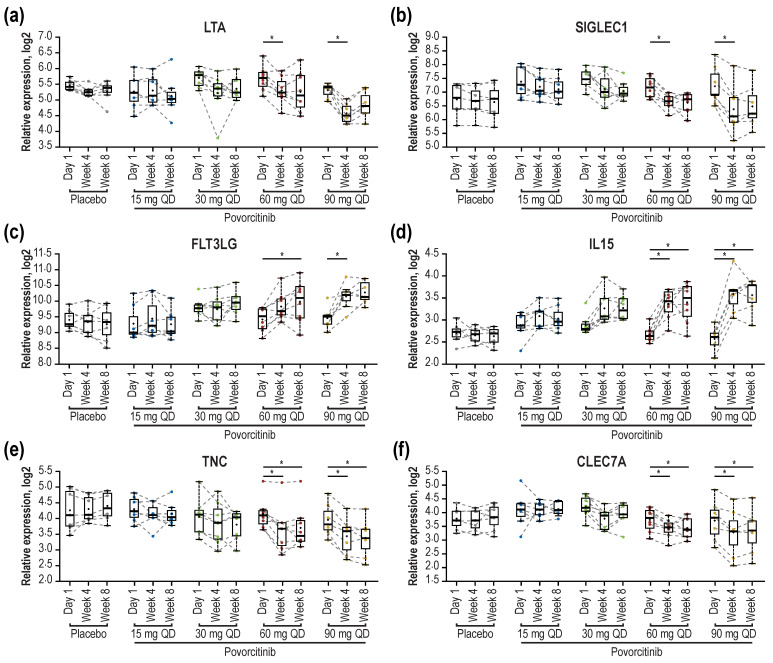
Modulation of circulating protein expression in blood samples over 8 weeks of treatment. (**a**) LTA, (**b**) SIGLEC1, (**c**) FLT3LG, (**d**) IL15, (**e**) TNC, and (**f**) CLEC7A. CLEC7A, C-type lectin domain containing 7A; |FCH|, absolute fold change; FDR, false discovery rate; FLT3LG, Fms-related receptor tyrosine kinase 3 ligand; IL, interleukin; LTA, lymphotoxin-alpha; SIGLEC1, sialic acid-binding Ig-like lectin 1; TNC, tenascin C. * FDR < 0.05. The cutoff for significance was FDR < 0.05 and |FCH| > 1.5.

Based on paired comparisons and the significance cutoff, a total of 33 differentially expressed proteins (DEPs) of interest were selected (6 upregulated and 27 downregulated proteins), representing the union of statistically significant upregulated and downregulated proteins for each treatment at Weeks 4 and 8 (FDR < 0.05 and absolute fold change [|FCH|] > 1.5). Modulation of these DEPs appeared to be dose dependent both at Week 4 (Figure 5a) and Week 8 (Figure 5b) and suggested a rapid onset of response, as these changes were generally observed as early as Week 4 and maintained over time. As has been previously reported [19], IL2RA was significantly reduced by Week 4 following treatment with 60 and 90 mg povorcitinib QD, with milder reductions observed in sera from patients treated with 15- or 30-mg doses. Furthermore, protein levels of lymphotoxin-alpha (LTA) and sialic acid-binding Ig-like lectin 1 (SIGLEC1) were significantly reduced by Week 4 following treatment with 60 and 90 mg povorcitinib QD, with milder reductions observed with 15- or 30-mg doses (Figure 6a,b). Conversely, levels of Fms-related receptor tyrosine kinase 3 ligand (FLT3LG; Figure 6c) and IL15 (Figure 6d) were increased after treatment with 60 and 90 mg povorcitinib QD. In addition to the DEPs identified above, tenascin C (TNC) and C-type lectin domain containing 7A (CLEC7A), previously shown to be upregulated in HS versus healthy controls [7], missed the analysis cutoff for DEP (|FCH| > 1.5 and FDR < 0.05) but were both significantly reduced based on FDR alone by Week 4 following treatment with 60 and 90 mg povorcitinib QD (Figure 6e,f). Other proteins that were differentially expressed in response to povorcitinib included CCL18, CLEC4D, GZMB, LAIR2, SH2D1A, and TNFRSF6B (Figure 5), whose corresponding genes were previously shown to be highly expressed in HS lesional (vs. non-lesional) and wounded (vs. non-wounded) skin [10]. Finally, treatment with povorcitinib led to the differential expression of CXCL10, GZMH, IL12B, IL2RA, and TNFSF11, whose corresponding genes were previously shown to be highly expressed in lesional skin from patients with HS compared with healthy controls [9].

## 3. Discussion

Hidradenitis suppurativa is a chronic, painful disease that can lead to permanent tissue damage and scarring, and it has a significant negative impact on quality of life [1]. There is a lack of effective treatment options for HS, which is likely attributable, at least in part, to a limited understanding of the underlying disease pathophysiology [3,4]. In this study, treatment with 30 mg povorcitinib QD was associated with the regulation of multiple markers of HS and inflammatory signaling in the transcriptome of skin biopsies. Based on gene signatures identified in previous studies, povorcitinib reversed gene expression associated with HS lesional skin, wounded skin, or the intersection of both [9,10]. Consistent with its proposed mechanism as a selective JAK1 inhibitor, povorcitinib resulted in the downregulation of multiple JAK/STAT–regulated transcripts (Figure 2b). We also observed reduced gene expression for pathways known to be elevated in HS lesions, suggesting that targeting JAK1 was modifying downstream inflammatory pathways. These included genes regulated by NFkB in response to TNF-α and genes regulated by TGF-β signaling. Moreover, we show treatment with povorcitinib modifying a previously identified HS disease signature [9], (99 of 128 genes were significantly reduced) revealing an overall shift in gene expression pattern away from the inflammatory processes associated with HS. Likewise, in a previous transcriptomic analysis, several genes associated with sweat gland function were reduced in HS compared with healthy skin [10]. Because functioning sweat glands are important for normal wound healing [22], the authors of that study postulated that dysfunctional sweat glands may contribute to the chronic nature of HS lesions [10]. Notably, we observed increases in several genes (*WIF1*, *KRT31*, *KRT77*, and *FOXA1)* associated with sweat gland development and function [23,24,25,26] after treatment with povorcitinib, suggesting that this process is also returning to normal in povorcitinib-treated HS skin.

Treatment with povorcitinib QD over 8 weeks was associated with a dose-dependent modulation of several proteins of interest, some of which have been previously reported to be differentially expressed in serum samples from patients with moderate-to-severe HS versus those from healthy controls [7]. For example, FLT3LG, which is inversely correlated with HS disease severity [7], increased after treatment with 60 and 90 mg povorcitinib QD. Additionally, expression of IL2RA, which can be induced by TNF signaling and is upregulated under chronic inflammatory conditions [27], was dose-dependently reduced in the sera of patients receiving povorcitinib. Finally, expression of LTA, a protein associated with chronic wounds [28]; SIGLEC1, an IFN-responsive protein restricted to monocytes and macrophages that suppresses antiviral innate immune response [29,30]; TNC, an extracellular matrix glycoprotein elevated in several inflammatory diseases [31,32,33]; and CLEC7A (or dectin-1), a protein involved in innate immune responses [34], were also all dose-dependently reduced. Together, these data corroborate findings from the clinical efficacy of povorcitinib as demonstrated in the phase 2 studies that showed a dose-dependent clinical improvement, with the proteomic analysis suggesting a rapid effect on protein expression [19]. HS clinical trials are typically 12 to 16 weeks in length, whereas patients were treated with povorcitinib for only 8 weeks in the current study. Nonetheless, a rapid change in protein expression (as early as the first assessment at Week 4) was seen. A similar rate of onset was observed in terms of clinical response (especially for International HS Severity Score System change from baseline) and improvements in HS symptoms (as assessed using the HS Quality of Life, worst skin pain numerical rating scale, and Dermatology Life Quality Index patient-reported outcome measures) [19].

These findings may also have implications for the identification of predictive biomarkers in HS. Few studies have investigated the role of biomarkers in predicting HS treatment response, and there are currently no validated clinical biomarkers for this purpose [35]. A prior RNA-seq analysis of HS lesions before 14 weeks of treatment with the anti–TNF-α antibody adalimumab revealed over 400 DEGs in pretreatment samples from patients who responded to therapy versus nonresponders, with responders generally showing a reduced inflammatory signature and more markers of skin regeneration at baseline [36]. Additionally, previous proteomic and biomarker studies have identified several potential immune-related serum markers of response to adalimumab, infliximab (anti–TNF-α antibody), and ustekinumab (anti–IL-12/IL-23) [37,38,39]. Notably, none of these previous studies examined treatment-related changes in markers at various time points, and the endpoints of these studies were Week 12 of treatment or later, hindering a direct comparison of response onset between povorcitinib and these biologics.

The study described herein was limited by the small sample size and unavailability of paired biopsies for all patients, which may affect the interpretability of our findings. Transcriptomic data were not available for time points between baseline and Week 8, nor from the 60 and 90 mg povorcitinib QD dose groups, which limits the detection of time- and dose-dependent effects of povorcitinib on DEGs. In addition, patients in the study were not stratified by disease severity, and this study had a short treatment duration (8 weeks) compared with previously reported HS clinical trials. In contrast, proteomic data were available for all dose groups and for both Week 4 and Week 8 timepoints. These data show that proteomic changes appeared to already plateau by 4 weeks, suggesting a rapid onset of action.

## 4. Materials and Methods

### 4.1. Study Design and Patients

Clinical samples were collected from two phase 2 studies of patients with moderate-to-severe HS [19]. Study 1 (NCT03569371; *n* = 10) was an open-label, single-arm trial in which all enrolled patients received oral povorcitinib 15 mg QD. Study 2 (NCT03607487; *n* = 35) was a randomized, placebo-controlled trial in which enrolled patients were randomized to receive oral povorcitinib 30 mg (*n* = 9), 60 mg (*n* = 9), or 90 mg (*n* = 8) or placebo (*n* = 9 [3 patients for each povorcitinib dose cohort]). Both studies included a treatment period of 8 weeks, followed by a safety follow-up period of approximately 4 weeks. A detailed description of patient demographics and baseline clinical characteristics for both study populations has been previously reported [19].

Serum was collected from all participants at baseline and Weeks 4 and 8 for proteomic analysis. Availability of skin biopsy samples for transcriptomic analysis was limited to 12 sample pairs (15 mg povorcitinib once daily [QD], *n* = 6; 30 mg povorcitinib QD, *n* = 4; placebo QD, *n* = 2).

### 4.2. Transcriptomic Analyses

RNA sequencing was conducted on frozen samples at the Beijing Genomics Institute using the Illumina HiSeq 4000 system (Illumina, Inc., San Diego, CA, USA), and sequences were aligned by using the Human Genome B38 library (National Center for Biotechnology Information) and the Gencode.V29 gene model (Gencode Project, EMBL-EBI). The fragments per kilobase of transcript per million mapped reads were generated, normalized, log2 transformed, and used for all downstream analyses.

Pathway enrichment was performed using Hallmark gene sets from the Molecular Signatures Database (MSigDB v7.2), which were generated using computational methodology and show coherent expression in well-defined biological states [21]. GSEA is a computational method that determines whether 2 biological states (e.g., phenotypes) are significantly different based on the expression of a predefined set of genes, with positive and negative enrichment scores indicating gene set enrichment at the top and bottom of the ranked list, respectively [40]. In this analysis, GSEA was used to evaluate the effects of 30 mg povorcitinib QD on a previously reported HS gene signature identified by comparing lesional HS skin versus healthy control skin [9], as well as data from two previously published public data sets describing transcriptomic signatures of inflammatory lesions versus unaffected non-lesional skin in patients with HS (3 mm biopsy samples; GSE72702) [39] and of wounded (3 mm punch biopsy) versus non-wounded axillary skin (GSE97615) [10,41]. The fold change from the paired *t* test of Week 8 versus Day 1 on samples from the 30 mg povorcitinib QD group was used to compile a ranked list of genes against which the Preranked function of GSEA was run, the results of which determined whether the collected signatures were statistically significantly enriched at either end of the ranking.

### 4.3. Proteomic Analyses

Blood samples were obtained at baseline, Week 4, and Week 8 from multiple dose groups (15 mg povorcitinib QD, *n* = 7; 30 mg povorcitinib QD, *n* = 8; 60 mg povorcitinib QD, *n* = 8; 90 mg povorcitinib QD, *n* = 7; placebo QD, *n* = 7). Proteomic data were evaluated using the Olink Normalized Protein eXpression (NPX) platform according to manufacturers’ instructions (Olink, Uppsala, Sweden; tests performed at Olink Analysis Service, Boston, MA, USA). The Olink NPX platform used in this study assessed 1104 protein assays from the following 12 Olink panels (92 proteins/panel): Organ Damage (v.3311), Inflammation (v.3021), Neuro Exploratory (v.3901), Development (v.3511), Cardiometabolic (v.3603), Oncology II (v.7004), Immune Response (v.3202), Neurology (v.8012), Cardiovascular III (v.6112), Cardiovascular II (v.5004), Cell Regulation (v.3702), and Metabolism (v.3403).

### 4.4. Statistical Analyses

For transcriptomic analyses, a paired *t* test was used to identify DEGs from Week 8 versus baseline between 15 mg and 30 mg povorcitinib QD groups. For proteomic analyses, a paired *t* test was used to identify DEPs from Week 4 versus Day 1 and Week 8 versus Day 1 for each treatment group. The cutoff for significance was an |FCH| > 1.5 and *p* < 0.05 for DEGs and FDR < 0.05 for DEPs.

## 5. Conclusions

In conclusion, based on findings observed in skin biopsies and blood of patients with moderate-to-severe HS, 8 weeks of treatment with the JAK1 inhibitor povorcitinib was associated with the reversal of a previously identified HS transcriptomic signature, as well as dose-dependent changes in a number of circulating proteins that may contribute to disease pathology. In agreement with the mechanism of action of povorcitinib, many observed changes involved pathways modulated by JAK/STAT signaling. Other biomarker changes seemed to reflect the clinical efficacy findings and therefore highlight the potential of JAK1 inhibition to modulate the underlying disease pathology in HS.

## Data Availability

Access to individual patient-level data is not available for this study. Information on Incyte’s clinical trial data sharing policy and instructions for submitting clinical trial data requests are available at: https://www.incyte.com/Portals/0/Assets/Compliance%20and%20Transparency/clinical-trial-data-sharing.pdf?ver=2020-05-21-132838-960 (accessed on 3 April 2023).

## Supplementary Materials

The following supporting information can be downloaded at: https://www.mdpi.com/article/10.3390/ijms24087185/s1.
