# Supplementary material for: Modulation of Disease-Associated Pathways in Hidradenitis Suppurativa by the Janus Kinase 1 Inhibitor Povorcitinib: Transcriptomic and Proteomic Analyses of Two Phase 2 Studies
Source: Int J Mol Sci. 2023 Apr 13;24(8):7185. doi: 10.3390/ijms24087185 (PMC10139090; doi:10.3390/ijms24087185)
Supplement: Supplementary file 1 [file ijms-24-07185-s001.zip › ijms-2254915-supplementary.pdf]

Modulation of Disease-Associated Pathways in Hidradenitis Suppurativa by the Janus Kinase 1 Inhibitor Povorcitinib: Transcriptomic and Proteomic Analyses of Two Phase 2 Studies

Huiqing Liu, Leandro L. Santos, Susan H. Smith

SUPPLEMENTARY MATERIAL

Table S1. Genes upregulated in HS lesional and wounded skin* and downregulated at Week 8 on 30 mg povorcitinib QD

Gene name	Gene description	Fold change	*P* value
ACER3	alkaline ceramidase 3	–1.6345	0.0441
ACSL4	acyl-CoA synthetase long chain family member 4	–1.5988	0.0222
ADAM12	ADAM metallopeptidase domain 12	–11.3031	0.0151
ADAMDEC1	ADAM like decysin 1	–6.9059	0.006
AIM2	absent in melanoma 2	–4.0243	0.0195
APOL6	apolipoprotein L6	–1.6956	0.0101
ARHGAP9	Rho GTPase activating protein 9	–1.7407	0.0159
ARRB2	arrestin beta 2	–1.7859	0.0167
B4GALT1	beta-1,4-galactosyltransferase 1	–1.8333	0.0301
BASP1	brain abundant membrane attached signal protein 1	–1.8224	0.0385
BCAT1	branched chain amino acid transaminase 1	–4.3055	0.013
C3AR1	complement C3a receptor 1	–2.3998	0.0223
CCL3	C-C motif chemokine ligand 3	–8.1517	0.036
CCND2	cyclin D2	–1.8101	0.0039
CCNYL1	cyclin Y like 1	–1.6231	0.0054
CCR1	C-C motif chemokine receptor 1	–2.0599	0.0342
CD14	CD14 molecule	–2.8617	0.0423
CD180	CD180 molecule	–2.3079	0.0155
CD38	CD38 molecule	–3.4444	0.009
CD53	CD53 molecule	–2.2345	0.0437
CD68	CD68 molecule	–2.8015	0.0482
CD79A	CD79a molecule	–6.3695	0.0088
CLEC7A	C-type lectin domain containing 7A	–1.8299	0.0305
CLIC4	chloride intracellular channel 4	–1.5323	0.044
CPPED1	calcineurin like phosphoesterase domain containing 1	–1.5538	0.0106
CTSB	cathepsin B	–2.4249	0.0401
CTSS	cathepsin S	–2.4367	0.0498
CYTH4	cytohesin 4	–1.9404	0.0117
DEFB103B	defensin beta 103B	–3.2352	0.0305

Gene name	Gene description	Fold change	*P* value
EAF2	ELL associated factor 2	–2.0332	0.0117
ELK3	ETS transcription factor ELK3	–1.6487	0.0084
ENTPD7	ectonucleoside triphosphate diphosphohydrolase 7	–1.8179	0.0345
EPHB2	EPH receptor B2	–2.1363	0.0308
EPSTI1	epithelial stromal interaction 1	–2.8264	0.0275
FBLIM1	filamin binding LIM protein 1	–1.7438	0.0379
FCGR2A	Fc fragment of IgG receptor IIa	–2.0098	0.0232
FCGR3A	Fc fragment of IgG receptor IIIa	–4.0163	0.00004933<br>2
FCRL5	Fc receptor like 5	–4.5066	0.0461
FKBP11	FKBP prolyl isomerase 11	–2.9842	0.0097
FPR3	formyl peptide receptor 3	–2.02	0.0441
GLIPR1	GLI pathogenesis related 1	–2.3	0.0391
GLIS3	GLIS family zinc finger 3	–2.9871	0.005
GNGT2	G protein subunit gamma transducin 2	–2.3745	0.0482
GPR180	G protein-coupled receptor 180	–1.6161	0.0244
HIF1A	hypoxia inducible factor 1 subunit alpha	–1.5888	0.0024
HM13	histocompatibility minor 13	–1.6778	0.0078
HSPA13	heat shock protein family A (Hsp70) member 13	–1.65	0.0419
IFI44	interferon induced protein 44	–2.0015	0.0292
IFNAR2	interferon alpha and beta receptor subunit 2	–2.4014	0.0431
IGFL1	IGF like family member 1	–10.6732	0.0091
IGSF6	immunoglobulin superfamily member 6	–2.7268	0.0076
IKBIP	IKBKB interacting protein	–2.1645	0.0354
IL10RA	interleukin 10 receptor subunit alpha	–1.5912	0.0418
IL1R1	interleukin 1 receptor type 1	–1.574	0.0151
IL24	interleukin 24	–3.8293	0.0259
ISG20	interferon stimulated exonuclease gene 20	–3.2504	0.0236
LCE3D	late cornified envelope 3D	–3.3618	0.0134
LCP2	lymphocyte cytosolic protein 2	–2.313	0.0201
LGALS3BP	galectin 3 binding protein	–1.5236	0.0151
LGMN	legumain	–1.7124	0.0371
LHFPL2	LHFPL tetraspan subfamily member 2	–3.3473	0.0253
LILRB4	leukocyte immunoglobulin like receptor B4	–3.9297	0.034
LIMK1	LIM domain kinase 1	–1.8275	0.0308
LIMS1	LIM zinc finger domain containing 1	–1.6769	0.0287
LMAN1	lectin, mannose binding 1	–1.6313	0.0085
LOXL2	lysyl oxidase like 2	–3.3653	0.0055
MAP4K4	mitogen-activated protein kinase kinase kinase kinase 4	–1.681	0.0095
MCHR1	melanin concentrating hormone receptor 1	–3.8003	0.0444
MICAL2	microtubule associated monooxygenase, calponin and LIM domain containing 2	–2.1772	0.0046
MLKL	mixed lineage kinase domain like pseudokinase	–1.5569	0.0484

Gene name	Gene description	Fold change	*P* value
MME	membrane metalloendopeptidase	–3.5278	0.028
MMP19	matrix metallopeptidase 19	–3.3869	0.0027
MS4A7	membrane spanning 4-domains A7	–2.593	0.0024
MYO1B	myosin IB	–1.6001	0.0427
NEK6	NIMA related kinase 6	–2.9387	0.0465
NID2	nidogen 2	–3.3054	0.0102
NRP2	neuropilin 2	–2.2141	0.0383
OAS2	2'-5'-oligoadenylate synthetase 2	–2.388	0.0177
OASL	2'-5'-oligoadenylate synthetase like	–3.6547	0.0003
PARP14	poly(ADP-ribose) polymerase family member 14	–1.6572	0.0004
PDLIM7	PDZ and LIM domain 7	–1.6725	0.0387
PDPN	podoplanin	–3.6451	0.0304
PHTF1	putative homeodomain transcription factor 1	–1.9296	0.024
PIK3CG	phosphatidylinositol-4,5-bisphosphate 3-kinase catalytic subunit gamma	–2.1048	0.0305
PIM2	Pim-2 proto-oncogene, serine/threonine kinase	–2.9058	0.0257
PLSCR1	phospholipid scramblase 1	–1.5503	0.0275
PRSS27	serine protease 27	–1.9619	0.0477
PXDN	peroxidasin	–2.7133	0.0308
RAB31	RAB31, member RAS oncogene family	–2.7477	0.0037
RAC2	Rac family small GTPase 2	–2.3494	0.0415
RASGRP3	RAS guanyl releasing protein 3	–2.0977	0.0289
RGS4	regulator of G protein signaling 4	–13.8938	0.0313
SEC24D	SEC24 homolog D, COPII coat complex component	–1.6053	0.027
SEL1L3	SEL1L family member 3	–2.7894	0.0073
SEMA6B	semaphorin 6B	–1.9958	0.0291
SERPINE1	serpin family E member 1	–3.0732	0.0255
SERPINH1	serpin family H member 1	–1.8582	0.0237
SH2D2A	SH2 domain containing 2A	–2.0535	0.0304
SKIL	SKI like proto-oncogene	–1.9601	0.0404
SLAMF7	SLAM family member 7	–2.4484	0.0418
SLC16A3	solute carrier family 16 member 3	–4.4785	0.0173
SLC19A1	solute carrier family 19 member 1	–1.8773	0.0262
SLC29A3	solute carrier family 29 member 3	–2.8006	0.0472
SLC35B4	solute carrier family 35 member B4	–1.5232	0.0046
SLC36A1	solute carrier family 36 member 1	–1.7275	0.0416
SLC39A7	solute carrier family 39 member 7	–1.6293	0.0217
SNX10	sorting nexin 10	–2.7172	0.024
SPI1	Spi-1 proto-oncogene	–1.9485	0.0442
SYNJ2	synaptojanin 2	–1.5958	0.0475
TBXAS1	thromboxane A synthase 1	–2.5839	0.0216
TDO2	tryptophan 2,3-dioxygenase	–15.6133	0.0135

Gene name	Gene description	Fold change	*P* value
TGFB1	transforming growth factor beta 1	–1.8075	0.0312
TLR1	toll like receptor 1	–1.8963	0.0424
TLR2	toll like receptor 2	–1.9007	0.0297
TMCO3	transmembrane and coiled-coil domains 3	–1.6121	0.0159
TMEM8A/ PGAP6	post-glycosylphosphatidylinositol attachment to proteins 6	–1.7611	0.0113
TNC	tenascin C	–3.1887	0.0251
TNFRSF21	TNF receptor superfamily member 21	–3.4002	0.018
TP53I3	tumor protein p53 inducible protein 3	–1.7805	0.0203
TREM2	triggering receptor expressed on myeloid cells 2	–10.9341	0.0092
TWSG1	twisted gastrulation BMP signaling modulator 1	–1.8653	0.0137
TXNDC5	thioredoxin domain containing 5	–1.7934	0.0023
UBE2J1	ubiquitin conjugating enzyme E2 J1	–1.6944	0.005
VNN3	vanin 3	–2.0548	0.0151
VOPP1	VOPP1 WW domain binding protein	–2.0079	0.0148
XAF1	XIAP associated factor 1	–2.3215	0.0239

HS, hidradenitis suppurativa; QD, once daily.

* Per Coates M, et al. *PLoS One*. 2019;14(5):e0216249.

Table S2. Genes downregulated in HS lesional and wounded skin* and upregulated at Week 8 on 30 mg povorcitinib QD

Gene name	Gene description	Fold change	*P* value
AADACL2	arylacetamide deacetylase like 2	2.2859	0.0218
ACADL	acyl-CoA dehydrogenase long chain	13.4549	0.0481
ACVR2A	activin A receptor type 2A	1.582	0.0188
ALDH3A2	aldehyde dehydrogenase 3 family member A2	2.1157	0.0157
ANKRD13B	ankyrin repeat domain 13B	2.0276	0.0247
ANO1	anoctamin 1	2.318	0.0411
BARD1	BRCA1 associated RING domain 1	2.2208	0.0187
BTC	betacellulin	4.7669	0.0344
C1orf21	chromosome 1 open reading frame 21	1.5075	0.029
CCDC146	coiled-coil domain containing 146	1.5971	0.0227
CDH22	cadherin 22	3.0328	0.014
CDH4	cadherin 4	6.4407	0.027
CHAD	chondroadherin	2.4	0.0106
CHP2	calcineurin like EF-hand protein 2	4.8181	0.0387
CTDSPL	CTD small phosphatase like	1.5678	0.015
CYP2J2	cytochrome P450 family 2 subfamily J member 2	2.5004	0.0207
CYP3A5	cytochrome P450 family 3 subfamily A member 5	3.4416	0.0473
CYP4F12	cytochrome P450 family 4 subfamily F member 12	3.5997	0.0077
DCST2	DC-STAMP domain containing 2	2.0008	0.037
DLK2	delta like non-canonical Notch ligand 2	1.8333	0.0135
EFNA3	ephrin A3	1.9635	0.0131
ELL3	elongation factor for RNA polymerase II 3	2.2292	0.0112
EPHB6	EPH receptor B6	2.7767	0.0447
EPHX2	epoxide hydrolase 2	2.4562	0.0037
FAM184B	family with sequence similarity 184 member B	1.7711	0.0039
GATA3	GATA binding protein 3	3.7841	0.038
GDPD2	glycerophosphodiester phosphodiesterase domain containing 2	3.4118	0.0263
GPC1	glypican 1	2.0849	0.0488
GPRASP2	G protein-coupled receptor associated sorting protein 2	1.6288	0.0134
HS3ST6	heparan sulfate-glucosamine 3-sulfotransferase 6	2.9727	0.0171
HSF2	heat shock transcription factor 2	1.5027	0.0428
ID4	inhibitor of DNA binding 4, HLH protein	2.3837	0.0061
IL20RB	interleukin 20 receptor subunit beta	1.9437	0.0293
KLF4	Kruppel like factor 4	2.2151	0.0199
KLK1	kallikrein 1	2.6567	0.0103
KRT31	keratin 31	4.1284	0.0137
KRT77	keratin 77	4.9531	0.0221
LONRF1	LON peptidase N-terminal domain and ring finger 1	2.0971	0.031
LRP4	LDL receptor related protein 4	2.4132	0.0388
LYPD6B	LY6/PLAUR domain containing 6B	2.6343	0.0499
MAPRE3	microtubule associated protein RP/EB family member 3	1.7221	0.0113
MAT1A	methionine adenosyltransferase 1A	4.7737	0.0071

Gene name	Gene description	Fold change	*P* value
MATN4	matrilin 4	18.9374	0.0053
MCF2L	MCF.2 cell line derived transforming sequence like	1.6721	0.0134
MFSD2A	major facilitator superfamily domain containing 2A	2.3374	0.0496
NDRG2	NDRG family member 2	1.8403	0.0394
NMNAT3	nicotinamide nucleotide adenylyltransferase 3	1.8265	0.0377
NPAS1	neuronal PAS domain protein 1	1.9023	0.0127
PGAP2	post-GPI attachment to proteins 2	1.6395	0.035
PHYHIP	phytanoyl-CoA 2-hydroxylase interacting protein	3.4178	0.0483
PLLP	plasmolipin	3.6563	0.0376
PNPLA7	patatin like phospholipase domain containing 7	1.5593	0.0048
PPFIA3	PTPRF interacting protein alpha 3	2.1823	0.032
PPM1J	protein phosphatase, Mg2+/Mn2+ dependent 1J	1.7156	0.0315
PRODH	proline dehydrogenase 1	4.7628	0.0278
RAB40C	RAB40C, member RAS oncogene family	1.9823	0.0343
RABL2B	RAB, member of RAS oncogene family like 2B	1.5741	0.0444
RARG	retinoic acid receptor gamma	2.0635	0.0421
RORC	RAR related orphan receptor C	2.9459	0.0008
RSPH1	radial spoke head component 1	2.4427	0.0093
SCEL	sciellin	2.8186	0.0105
SGSM1	small G protein signaling modulator 1	5.3227	0.0132
SOX6	SRY-box transcription factor 6	3.0954	0.0312
SP8	Sp8 transcription factor	7.2163	0.0202
STAR	steroidogenic acute regulatory protein	1.9417	0.0091
SYT8	synaptotagmin 8	4.9488	0.0133
TNFRSF25	TNF receptor superfamily member 25	1.683	0.0326
TNNI2	troponin I2, fast skeletal type	1.9221	0.0141
TNNT2	troponin T2, cardiac type	2.5694	0.0154
TRIM45	tripartite motif containing 45	2.193	0.0494
WEE1	WEE1 G2 checkpoint kinase	2.9984	0.028
WFIKKN1	WAP, follistatin/kazal, immunoglobulin, kunitz and netrin domain containing 1	3.508	0.0318
WIF1	WNT inhibitory factor 1	18.6288	0.024
YBX2	Y-box binding protein 2	8.1741	0.0053
ZDHHC11	zinc finger DHHC-type containing 11	2.8549	0.0433

HS, hidradenitis suppurativa; QD, once daily.

* Per Coates M, et al. *PLoS One*. 2019;14(5):e0216249.

Figure S1. Identification of gene signatures from HS lesional and wounded skin for enrichment analysis*

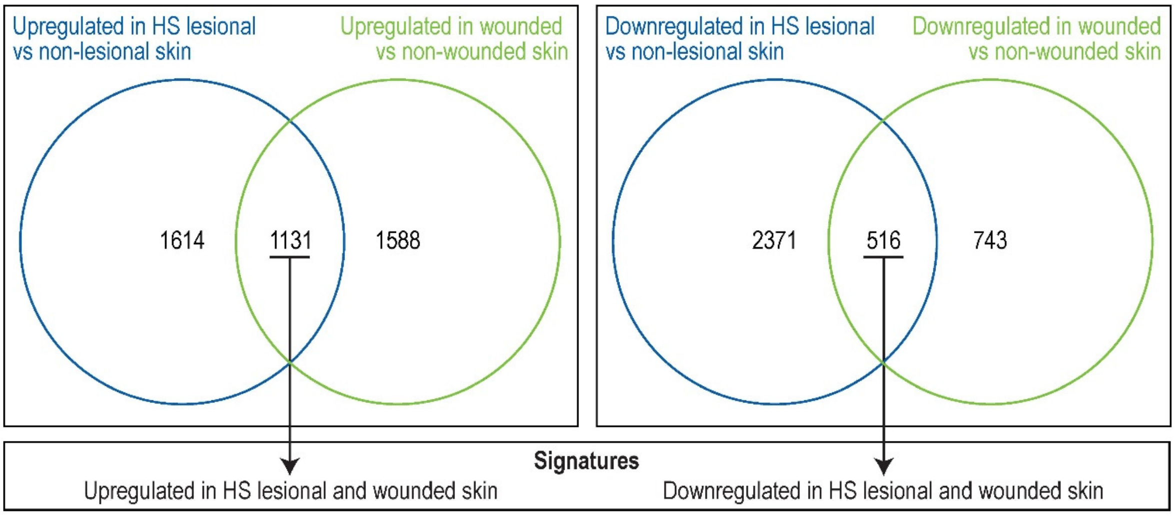

HS, hidradenitis suppurativa.
* Per Coates M, et al. *PLoS One*. 2019;14(5):e0216249.

Figure S2. GSEA and enrichment plots showing signature reversal of (a) upregulated genes in both HS lesional and wounded skin and (b) downregulated genes in both HS lesional and wounded skin after 8 weeks of treatment with 30 mg povorcitinib QD*

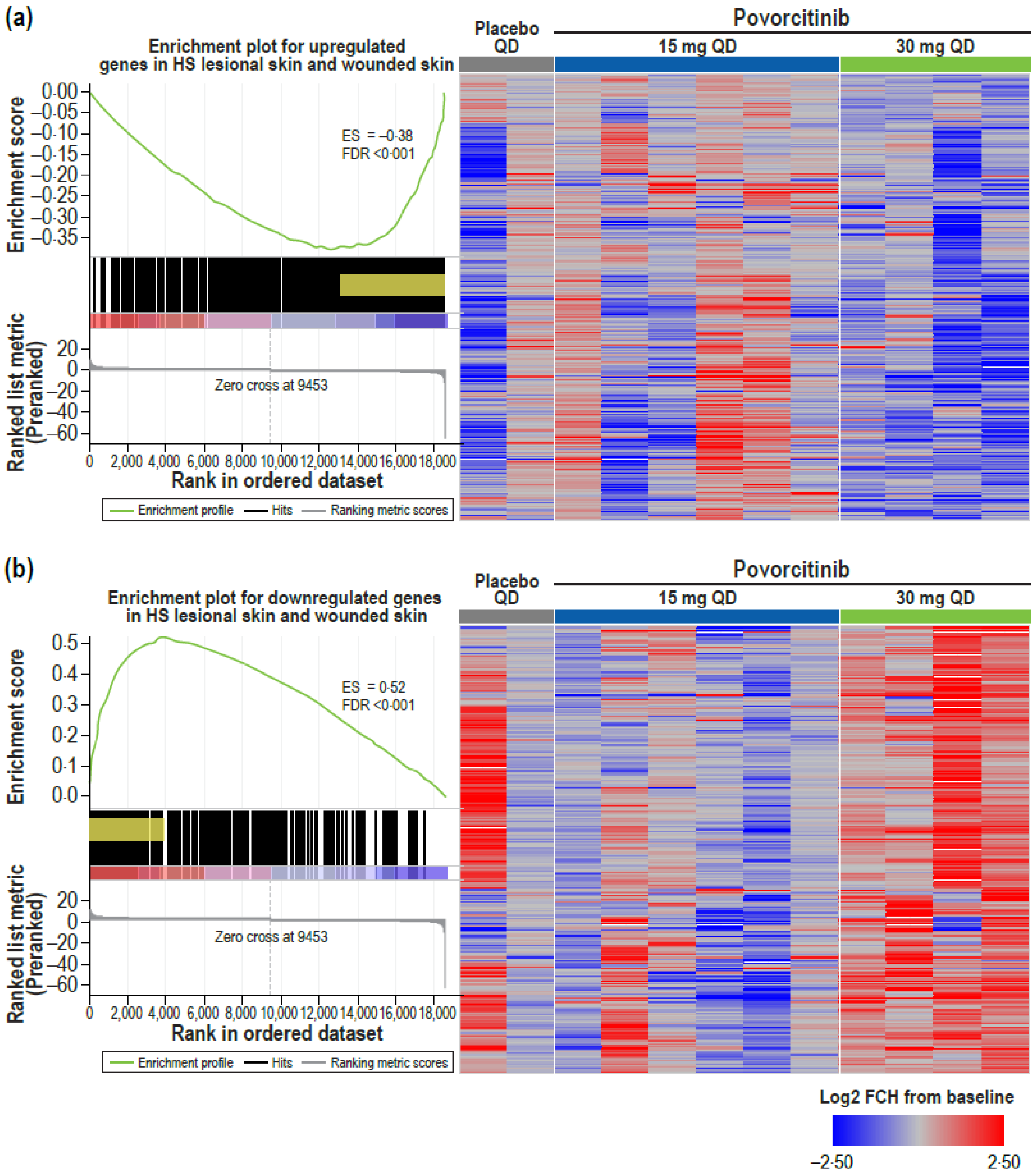

ES, enrichment score; FCH, fold change; FDR, false discovery rate; GSEA, gene set enrichment analysis; HS, hidradenitis suppurativa; QD, once daily.

* Each plot represents a case (30 mg povorcitinib QD treatment) and GSEA data based on previously described HS gene signatures: Coates M, et al. *PLoS One*. 2019;14(5):e0216249 ([a], HS-high and wounded-high; [b], HS-low and wounded-low). Genes displayed in each heatmap are marked in yellow in the corresponding GSEA plot.
